# Metabolomics Reveal Potential Natural Substrates of AcrB in Escherichia coli and Salmonella enterica Serovar Typhimurium

**DOI:** 10.1128/mBio.00109-21

**Published:** 2021-03-30

**Authors:** Xuan Wang-Kan, Giovanny Rodríguez-Blanco, Andrew D. Southam, Catherine L. Winder, Warwick B. Dunn, Alasdair Ivens, Laura J. V. Piddock

**Affiliations:** a Antimicrobials Research Group, Institute of Microbiology and Infection, College of Medical and Dental Sciences, The University of Birmingham, Birmingham, United Kingdom; b School of Biosciences, The University of Birmingham, Birmingham, United Kingdom; c Phenome Centre Birmingham, The University of Birmingham, Birmingham, United Kingdom; d Institute of Metabolism and Systems Research, University of Birmingham, Birmingham, United Kingdom; e Centre for Immunity, Infection and Evolution, University of Edinburgh, Edinburgh, United Kingdom; Indiana University Bloomington

**Keywords:** AcrAB, AcrAB-TolC, physiological substrates, efflux, *Enterobacterales*, *Escherichia coli*, *Salmonella*, drug efflux, efflux pumps

## Abstract

In the fight against antibiotic resistance, drugs that target resistance mechanisms in bacteria can be used to restore the therapeutic effectiveness of antibiotics. The multidrug resistance efflux complex AcrAB-TolC is the most clinically relevant efflux pump in *Enterobacterales* and is a target for drug discovery. Inhibition of the pump protein AcrB allows the intracellular accumulation of a wide variety of antibiotics, effectively restoring their therapeutic potency. To facilitate the development of AcrB efflux inhibitors, it is desirable to discover the native substrates of the pump, as these could be chemically modified to become inhibitors. We analyzed the native substrate profile of AcrB in Escherichia coli MG1655 and Salmonella enterica serovar Typhimurium SL1344 using an untargeted metabolomics approach. We analyzed the endo- and exometabolome of the wild-type strain and their respective AcrB loss-of-function mutants (AcrB D408A) to determine the metabolites that are native substrates of AcrB. Although there is 95% homology between the AcrB proteins of *S.* Typhimurium and E. coli, we observed mostly different metabolic responses in the exometabolomes of the *S.* Typhimurium and E. coli AcrB D408A mutants relative to those in the wild type, potentially indicating a differential metabolic adaptation to the same mutation in these two species. Additionally, we uncovered metabolite classes that could be involved in virulence of *S.* Typhimurium and a potential natural substrate of AcrB common to both species.

## Abstract (precis)

Multidrug-resistant Gram-negative bacteria pose a global threat to human health. The AcrB efflux pump confers inherent and evolved drug resistance to *Enterobacterales*, including Escherichia coli and Salmonella enterica serovar Typhimurium.

## INTRODUCTION

As an increasing number of microorganisms develop resistance to antibiotics, the efficacy of antibiotic treatment is compromised, and new antibiotics to treat infections are needed (1). Aside from the development of new antibiotics, drugs that can restore the effectiveness of an antibiotic are also a strategy for tackling drug-resistant infections (2, 3). The AcrAB-TolC multidrug resistance (MDR) efflux complex and its homologues in Gram-negative bacteria confer resistance to a wide variety of antibiotics. AcrAB-TolC confers antibiotic resistance by expelling antibiotics and other compounds from the bacterial cell, giving rise to MDR bacteria, as drugs cannot accumulate and reach their intracellular targets (4, 5). To reverse antibiotic resistance, the use of efflux inhibitors that target AcrB, the transporter component of the AcrAB-TolC complex, and prevent efflux have been proposed. AcrB is highly conserved among *Enterobacterales* and is functionally similar to homologous pumps in other species, such as MexB of Pseudomonas aeruginosa and AdeJ and AdeB of Acinetobacter baumannii (5, 6); making it an attractive target for the development of efflux inhibitors. Although it is well known that AcrB exports a wide range of antibiotics, the native bacterially derived substrates of this pump remain unknown. Identification of the physiological substrates of AcrB will provide an insight into the biological role of this pump and could facilitate the design of efflux inhibitors (7, 8), as the structure of natural substrates could form the basis for new inhibitors.

In addition to their role in drug resistance, efflux pumps from the resistance-nodulation-cell division transporter (RND) family are of industrial relevance. Overexpression of RND pumps can be exploited to enhance the bioproduction of multiple industrially relevant compounds, as they increase the tolerance of the producer strain to the compound of interest. Examples of these are biofuels, terpenes, and free fatty acids in Escherichia coli (9–13) and toluene and propranolol in Pseudomonas putida (14, 15). Knowledge about the natural substrates of AcrB will further help define which other industrially relevant compounds could be exported via the pump.

In this study, we used an untargeted metabolomics approach to analyze the physiological substrates of AcrB from two members of the *Enterobacterales* family: Escherichia coli MG1655 and Salmonella enterica serovar Typhimurium SL1344 (here referred to as *S.* Typhimurium). The AcrB protein of these two species share 95% protein sequence identity. Although closely related phylogenetically, the physiology and metabolism of these two species are different (16, 17). Using exemplar E. coli and *S.* Typhimurium strains allowed us to explore the physiological substrates of AcrB in bacteria with different metabolic phenotypes. Our study showed that the native substrate profile of AcrB is influenced by the genetic traits and metabolic phenotype of each species as well as the environmental conditions in which the bacteria grow. Of particular interest were three metabolite classes that may be involved in virulence of *S.* Typhimurium and one metabolite class that appears to be a common substrate of AcrB in both species.

## RESULTS

### In minimal medium, loss of AcrB function produces significant changes in the endometabolome but not in the exometabolome of E. coli.

To study changes in the export of metabolites by AcrB in E. coli, a mutant that lacked AcrB efflux function was constructed in E. coli MG1655. A single nucleotide polymorphism (SNP) was inserted in the chromosome of MG1655, which translated into AcrB D408A (see Fig. S1A in the supplemental material). Similar to that we previously reported for an *S.* Typhimurium SL1344 AcrB D408A mutant (18), expression of AcrB in the E. coli mutant was comparable to that in its parental wild-type strain (Fig. S1B). In addition, the mutant showed no growth deficiencies in LB broth or morpholinepropanesulfonic acid (MOPS) minimal medium (see Fig. S2) but demonstrated decreased efflux activity as shown by increased susceptibility to substrates of AcrAB-TolC (lower MICs of antibiotics) (Table S1) and increased accumulation of the dye Hoescht 33342 (Fig. S3A). However, unlike the *S.* Typhimurium *acrB* mutants (18), neither the Δ*acrB* nor the AcrB D408A E. coli mutant had altered efflux of ethidium bromide compared against the parental MG1655 strain (Fig. S3B).

10.1128/mBio.00109-21.1 FIG S1 Whole-genome sequencing (A) and Western blotting of AcrB (B) of the E. coli MG1655 AcrB D408A mutant. (A) Mutations found in the parental MG1655 strain (and consequently in its AcrB D408A mutant) used in this study and their position in the chromosome of the MG1655 wild-type strain used in this study are shown on the left. On the right, whole-genome sequencing (WGS) data visualization of *acrAB* in Artemis is shown. Each blue and green horizontal line represents a mapped read. The region corresponding to the *acrB* gene is highlighted in red, and the SNP that translates into the D408A substitution in the AcrB protein is shown as a red vertical line that goes across all mapped reads. (B) The concentration of AcrB relative to the concentration of RNA polymerase is shown. The intensity of the bands was determined using ImageJ. Wild type was designated 100% expression of AcrB, the percentage of AcrB expression in the Δ*acrB* and the AcrB D408A mutants were calculated based on the wild-type strain. Download FIG S1, TIF file, 2.7 MB. Copyright © 2021 Wang-Kan et al. 2021 Wang-Kan et al. https://creativecommons.org/licenses/by/4.0/ This content is distributed under the terms of the Creative Commons Attribution 4.0 International license.

10.1128/mBio.00109-21.2 FIG S2 Growth curves and generation times of E. coli MG1655 and its *acrB* mutants. Growth curve and generation times ± standard deviations of E. coli strains in LB broth (A) and MOPS minimal medium (B) are presented. Growth curves represent the means from three biological replicates. Generation times were calculated during exponential growth phase. Download FIG S2, TIF file, 0.3 MB. Copyright © 2021 Wang-Kan et al. 2021 Wang-Kan et al. https://creativecommons.org/licenses/by/4.0/ This content is distributed under the terms of the Creative Commons Attribution 4.0 International license.

10.1128/mBio.00109-21.3 FIG S3 Accumulation of Hoechst 33342 and efflux of ethidium bromide in E. coli MG1655 and its *acrB* mutants. (A) Accumulation kinetics and final fold change in accumulation of Hoechst 33342. (B) Efflux kinetics and final fold change in efflux of ethidium bromide. *, *P* < 0.05 between two groups, compared by Student’s *t* test with Welch’s correction; N.S, no significant difference between two groups. Download FIG S3, TIF file, 0.5 MB. Copyright © 2021 Wang-Kan et al. 2021 Wang-Kan et al. https://creativecommons.org/licenses/by/4.0/ This content is distributed under the terms of the Creative Commons Attribution 4.0 International license.

10.1128/mBio.00109-21.4 TABLE S1 MICs of AcrB substrates for E. coli MG1655 and its respective *acrB* mutants. CIP, ciprofloxacin; NAL, nalidixic acid; NOV, novobiocin; CHL, chloramphenicol; TET, tetracycline; MIN, minocycline; FUS, fusidic acid; OX, oxacillin; ERY, erythromycin; ACR, acriflavine; ETBR, ethidium bromide; RHO, rhodamine 6G. Agar dilution MICs were determined four times. Mode values are shown. Download Table S1, DOCX file, 0.01 MB. Copyright © 2021 Wang-Kan et al. 2021 Wang-Kan et al. https://creativecommons.org/licenses/by/4.0/ This content is distributed under the terms of the Creative Commons Attribution 4.0 International license.

To explore the metabolites exported by AcrB, the exometabolome (extracellular metabolome) of the E. coli AcrB D408A mutant was compared against that of its parental wild-type strain. Samples were obtained from stationary-phase cultures grown in MOPS minimal medium. To differentiate between exometabolomic changes caused by decreased export of metabolites from those caused by decreased intracellular synthesis of the metabolite, the endometabolome (intracellular metabolome) of both strains was extracted and analyzed in parallel. MOPS minimal medium was chosen for this experiment as, except for glucose added as carbon source, it does not contain any metabolites (19). Therefore, all metabolites observed in the exo/endometabolome are derived from *de novo* synthesis, and any changes observed in the exometabolome would be a consequence of differential efflux and not influx of nutrients.

For statistical robustness, ten independent cultures of each strain were processed. Under the conditions tested, no statistically significant differences in metabolite content were observed when the exometabolome of the mutant was compared against that of its parental strain. However, analysis of the endometabolome showed a significant (*P* < 0.05) change in the relative amounts of eight metabolites between the E. coli wild type and the AcrB D408A mutant, with a greater than 20% change in their relative concentration. It is important to note that the annotation of single metabolites from liquid chromatography-mass spectrometry (LC-MS) untargeted metabolomics is putative, as one peak can be identified as multiple metabolites that belong to the same class. Therefore, changes in metabolite classes are easier to identify and more reproducible than those in single metabolites. Hence, we primarily use metabolite classes to refer to changes in the content of the metabolomes.

An overall change in sphingolipid metabolism (KEGG pathway 00600) was observed in the E. coli AcrB D408A mutant, represented by higher concentrations of sphinganine and sphingosine in the mutant endometabolome. Four metabolites identified as oxidized fatty acids (structures similar to prostaglandins, leukotrienes, lipoxins, and thromboxanes) were present at a higher concentration in the mutant endometabolome (Table 1; see also Table S2), suggesting that this metabolite class accumulated in E. coli in the absence of AcrB-mediated efflux. However, as so few oxidized fatty acid metabolites were significantly different between the metabolomes, pathway enrichment analysis was unable to show this metabolite class as being significantly enriched.

**TABLE 1:** List of metabolites with significantly changed relative concentration between the endometabolome of the wild-type E. coli MG1655 and its AcrB D408A mutant in MOPS minimal medium

Metabolite/metabolite class^*a*^	Fold change (AcrB D408A/wild-type) or comment
Higher concn in the endometabolome of the E. coli wild-type strain	
Lysophosphatidic acid (15:0)/lysophosphatidylglycerol (13:0)	Only detected in the wild-type strain
Higher concn in the endometabolome of the E. coli AcrB D408A mutant	
C_16_ Sphinganine	1.24
Oxidized fatty acid	1.36
Oxidized fatty acid	1.45
Aspartyl-tryptophan	1.51
Oxidized fatty acid	1.59
Oxidized fatty acid	Only detected in the AcrB D408A mutant
Sphingosine	Only detected in the AcrB D408A mutant

a Multiple metabolite identities were assigned to each feature based on its mass and retention times. Each possible identity is separated by a slash (/). For the complete list of annotations, refer to Table S2 in the supplemental material.

10.1128/mBio.00109-21.5 TABLE S2 Extended list of metabolites with significantly changed relative concentration between the endometabolomes of the wild-type E. coli MG1655 and its AcrB D408A mutant in MOPS minimal medium. Download Table S2, XLSX file, 0.01 MB. Copyright © 2021 Wang-Kan et al. 2021 Wang-Kan et al. https://creativecommons.org/licenses/by/4.0/ This content is distributed under the terms of the Creative Commons Attribution 4.0 International license.

### In metabolite-rich medium, loss of AcrB efflux function causes significant changes in the exometabolome of E. coli.

An absence of significant changes in the metabolite content of the exometabolome comparison between the E. coli strains was an unexpected result. A hypothesis was that in a minimal medium such as MOPS, most synthesized metabolites were retained inside the cell for other biological processes, because the MOPS medium only contains one metabolite, glucose, which will be used as the carbon source to synthesize all intracellular metabolites required. This would decrease the diversity and concentration of metabolites exported by E. coli, resulting in the absence of detectable changes between the AcrB D408A mutant and its parental strain. In a metabolite-rich medium, such as LB broth, a number of essential metabolites are provided in the medium and do not need to be synthesized in the cell. Therefore, if LB broth was used, the abundance of nutrients may favor efflux of metabolites, increasing the diversity and concentration of these in the exometabolome.

To explore this hypothesis, the exometabolome of E. coli MG1655 grown in metabolite-containing LB broth was prepared and analyzed. A total of 11 metabolites were significantly different between the exometabolome of the wild-type E. coli and its AcrB D408A mutant. Six of the 11 metabolites were detected at a higher concentration in the wild-type exometabolome relative to that in the mutant (Table 2; see also Table S3). These metabolites are likely to be exported by AcrB, as loss of AcrB-mediated efflux decreased their concentration in the exometabolome and is discussed below. Interestingly, the remaining five metabolites were found at a higher concentration in the mutant exometabolome than in the wild-type strain (Table 2), suggesting that losing AcrB efflux activity increased the biosynthesis of these metabolites or their export via other pumps. Both groups contained porphyrin-derived metabolites and polyamines. However, an oxidized fatty acid was found among the metabolites that were likely to be exported by AcrB (increased concentration in the wild type). In contrast, the relative concentration of a nucleotide was increased in the exometabolome of the E. coli AcrB D408A mutant, suggesting that lack of AcrB-mediated efflux affected the export of this metabolite through other pumps or its biosynthesis.

**TABLE 2:** List of metabolites with significantly changed relative concentration between the exometabolome of the wild-type E. coli MG1655 and its AcrB D408A mutant in LB broth

Metabolite/metabolite class^*a*^	Fold change (AcrB D408A/wild-type) or comment
Higher concn in the exometabolome of the E. coli wild-type strain	
Vitamin D metabolite	Only detected in the wild-type strain
Oxidized fatty acid	0.49
Heptacarboxylporphyrin/pseudouroporphyrin	0.49
Methionyl-methionine	0.47
Isoputreanine	0.44
Isopropyl beta-d-glucoside	0.43
Higher concn in the exometabolome of the E. coli AcrB D408A mutant	
2′-Deoxyguanosine 5′-monophosphate/adenosine monophosphate	2.03
Glutamyl-threonine/threoninyl-glutamate	2.10
Heptacarboxylporphyrin I/hydroxypropionic porphyrin III/pseudouroporphyrin	2.20
5,6,7,8-Tetrahydro-2,4-dimethylquinoline	2.80
N5-Hexanoylspermidine	Only detected in the AcrB D408A mutant

a Multiple metabolite identities were assigned to each feature based on its mass and retention times. Each possible identity is separated by a slash (/). For the complete list of annotations, refer to Table S3.

10.1128/mBio.00109-21.6 TABLE S3 Extended list of metabolites with significantly changed relative concentration between the exometabolomes of the wild-type E. coli MG1655 and its AcrB D408A mutant in LB broth. Download Table S3, XLSX file, 0.01 MB. Copyright © 2021 Wang-Kan et al. 2021 Wang-Kan et al. https://creativecommons.org/licenses/by/4.0/ This content is distributed under the terms of the Creative Commons Attribution 4.0 International license.

### In minimal medium, loss of AcrB-mediated efflux only modified the endometabolome of *S.* Typhimurium, whereas the exometabolome was only modified in metabolite-rich medium.

To determine if our observations were unique to E. coli, the endo- and exometabolome of *S.* Typhimurium SL1344 and its AcrB D408A mutant grown in MOPS minimal medium were analyzed. Similar to the observations with E. coli, no significant differences were observed between the *S.* Typhimurium wild type and its AcrB D408A mutant exometabolome. Likewise, significant changes in seven metabolites were observed in the intracellular metabolome of the *S.* Typhimurium AcrB D408A mutant. A metabolite putatively identified as *N*,*N*-dimethylsphing-4-enine, related to sphingolipid metabolism, was present at a lower concentration in the wild type than in the mutant strain. Two oxidized fatty acids were also present at higher or lower concentrations in the wild-type strain (Table 3; see Table S4).

**TABLE 3:** List of metabolites with significantly changed relative concentration between the endometabolome of the wild-type *S.* Typhimurium SL1344 and its AcrB D408A mutant in MOPS minimal medium

Metabolite/metabolite class^*a*^	Fold change (AcrB D408A/wild-type) or comment
Higher concn in the endometabolome of the *S.* Typhimurium wild-type strain	
Glutaminylphenylalanine/phenylalanylglutamate	Only detected in the wild-type strain
Fatty acid (NO_2_-conjugated linoleic acid)	0.73
Oxidized fatty acid	0.73
Higher concn in the endometabolome of the *S.* Typhimurium AcrB D408A mutant	
*N*,*N*-Dimethylsphing-4-enine	1.21
Tetrahydrogeranylgeranyl diphosphate	1.27
Hydroxypentanoate/hydroxyisopentanoate	1.92
Dihydroxyeicosanoic acid	Only detected in the AcrB D408A mutant

a Multiple metabolite identities were assigned to each feature based on its mass and retention times. Each possible identity is separated by a slash (/). For the complete list of annotations, refer to Table S4.

10.1128/mBio.00109-21.7 TABLE S4 Extended list of metabolites with significantly changed relative concentration between the endometabolomes of the wild-type *S.* Typhimurium SL1344 and its AcrB D408A mutant in MOPS minimal medium. Download Table S4, XLSX file, 0.01 MB. Copyright © 2021 Wang-Kan et al. 2021 Wang-Kan et al. https://creativecommons.org/licenses/by/4.0/ This content is distributed under the terms of the Creative Commons Attribution 4.0 International license.

In LB broth, a total of 178 metabolites were significantly different between the exometabolome of the wild type and that of the AcrB D408A mutant of *S.* Typhimurium. This was considerably more than the number of metabolites found in the exometabolome of E. coli and is discussed below. Of the 178 metabolites in *S.* Typhimurium, 102 were found at a significantly higher concentration in the wild-type exometabolome than in that of the AcrB D408A mutant. These could be considered possible substrates of AcrB. The remaining 76 metabolites were found in a higher concentration in the exometabolome of the AcrB D408A mutant (see Table S5). Metabolites were grouped into 17 classes (Table 4). Some metabolite classes demonstrated a trend of having similar numbers of metabolites present at a higher concentration in the exometabolome of the AcrB D408A mutant and in the wild-type exometabolome (type 1). Other metabolite classes demonstrated that the majority of metabolites with different concentrations were observed only in the wild-type (type 2) or the AcrB D408A mutant (type 3) exometabolome. Examples of classes in type 1 were peptides, fatty acids and related metabolites, and oxidized fatty acids as well as glycerophospholipids and associated metabolism.

**TABLE 4:** Number of metabolites found in higher or lower concentrations in the exometabolome of the *S.* Typhimurium AcrB D408A mutant relative to its parental wild-type strain in LB broth

Metabolite class	Total no. of metabolites	No. with higher concn in the exometabolome from:	
		Wild-type strain	AcrB D408A mutant
Peptide	21	13	8
Tryptophan/phenylalanine metabolism	16	12	4
Fatty acid and related metabolite	14	9	5
Sterol and steroid metabolism	13	4	9
Lysoglycerophospholipid	12	8	4
Oxidized fatty acids	11	4	7
Glycerophospholipid and associated metabolism	10	5	5
Acyl carnitine	8	6	2
Acyl amino acid	5	1	4
Polyamine	3	3	0
Purine and pyrimidine metabolism	3	0	3
			
Total	178	102	76

10.1128/mBio.00109-21.8 TABLE S5 List of metabolites with significantly changed relative concentration between the exometabolomes of the wild-type *S.* Typhimurium SL1344 and its AcrB D408A mutant in LB broth. Download Table S5, XLSX file, 0.02 MB. Copyright © 2021 Wang-Kan et al. 2021 Wang-Kan et al. https://creativecommons.org/licenses/by/4.0/ This content is distributed under the terms of the Creative Commons Attribution 4.0 International license.

The presence of these metabolites in similar numbers in the exometabolomes of the wild type and AcrB D408A mutant suggested that although the metabolite class could contain native substrates of AcrB, they could also be exported by other transporters in *S.* Typhimurium SL1344. In type 2, the tryptophan/phenylalanine class contained 16 statistically significant metabolites, of which 12 were present at a higher concentration in the exometabolome of the wild-type strain. Similarly, polyamines (three metabolites), acyl carnitines (six metabolites), and lysoglycerophospholipids (eight metabolites) were predominantly observed at a higher concentration in the wild-type exometabolome. In type 3, purine and pyrimidine metabolism (three metabolites), acyl amino acids (four metabolites), and sterol and steroid metabolites (nine metabolites) were predominantly found at a higher concentration in the mutant exometabolome (Table 4; Table S5).

### Intracellular metabolic changes caused by loss of AcrB efflux are driven primarily by the physiology of the microorganism.

**FIG 1:**
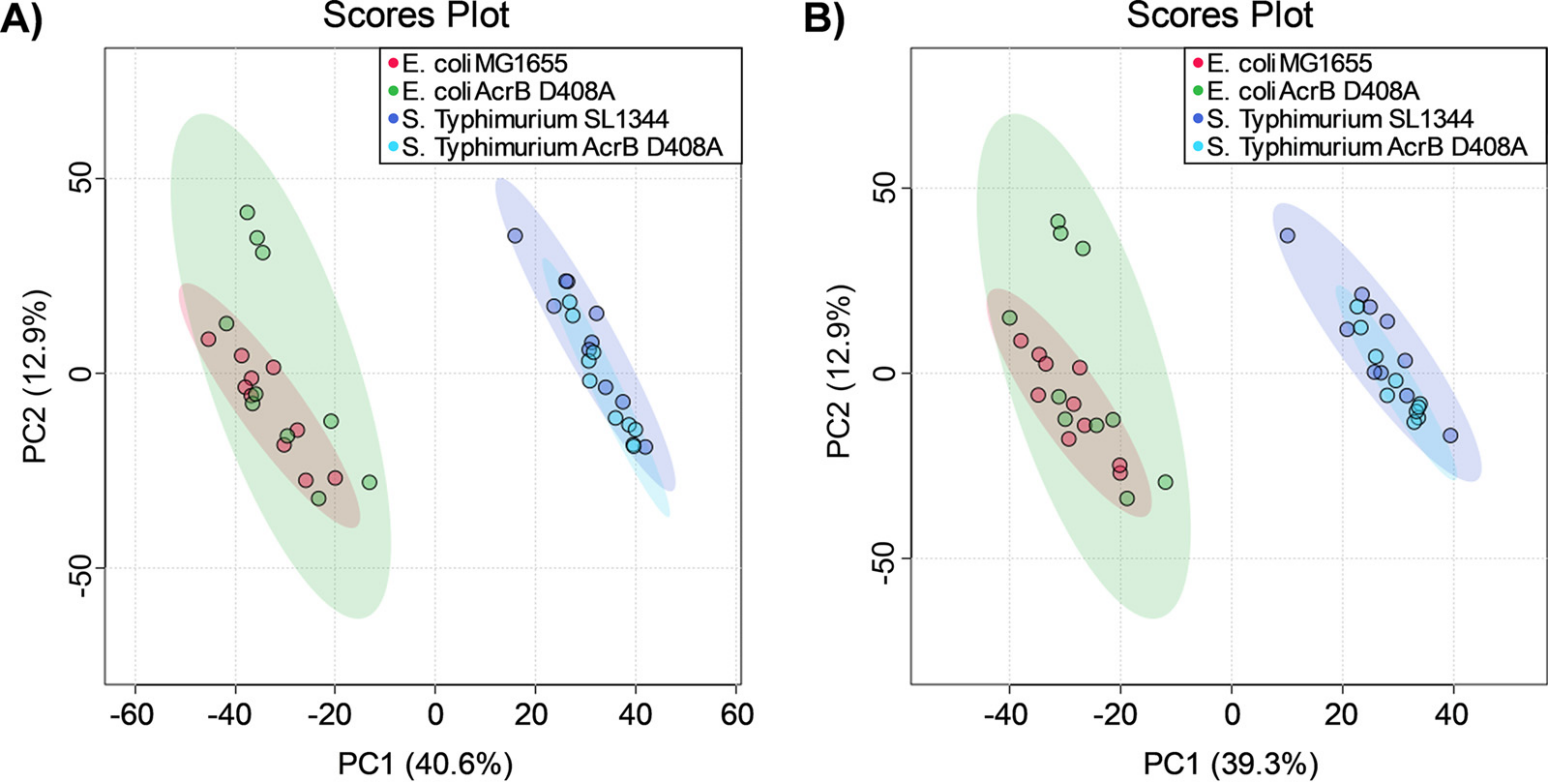
Principal-component analysis of the endometabolome of the wild types and AcrB D408A mutants of E. coli MG1655 and *S.* Typhimurium SL1344 in MOPS minimal medium. Endometabolomes were analyzed by hydrophilic interaction chromatography (HILIC)-MS in positive mode (A) and negative mode (B). Each dot represents a biological replicate of each strain.

An interesting observation was the abundance of sphingolipid and fatty acid metabolites in the endometabolome of the E. coli and *S.* Typhimurium AcrB D408A mutants grown in MOPS minimal medium. Although the same metabolite classes were observed to be perturbed, the metabolites perturbed in each organism were different as shown by different *m/z* values and retention times of the respective peaks. Therefore, we hypothesized that there was little metabolic overlap between the endometabolome of the two organisms in the absence of AcrB-mediated efflux. To assess this, a principal-component analysis (PCA) was carried out (Fig. 1). The analysis showed that E. coli and *S.* Typhimurium species (wild type and AcrB D408A mutant combined) formed two separate clusters, indicating that they are metabolically different, while the *acrB* mutants clustered tightly with their respective wild-type strain. These results suggest that the major intracellular metabolic differences were associated with species rather than efflux function.

During the analysis of the extracellular metabolome of *S. Typhimurium* and E. coli in LB broth, we observed that the concentrations of a large number of metabolites were significantly affected by loss of AcrB efflux in *S.* Typhimurium (178 metabolites). This was approximately 16 times more than the number of altered metabolites observed in E. coli (11 metabolites). This further suggested that the impact of loss of AcrB efflux function in the two strains was different. To explore this, RNA sequencing was carried out on E. coli MG1655 and *S.* Typhimurium SL1344 and their respective AcrB D408A mutants. The cultures were prepared in LB broth, and RNA was extracted from stationary-phase cultures to match the metabolomic analyses. Gene transcription patterns between these two microorganisms were compared by clusters of orthologous groups (COG) proteins.

**FIG 2:**
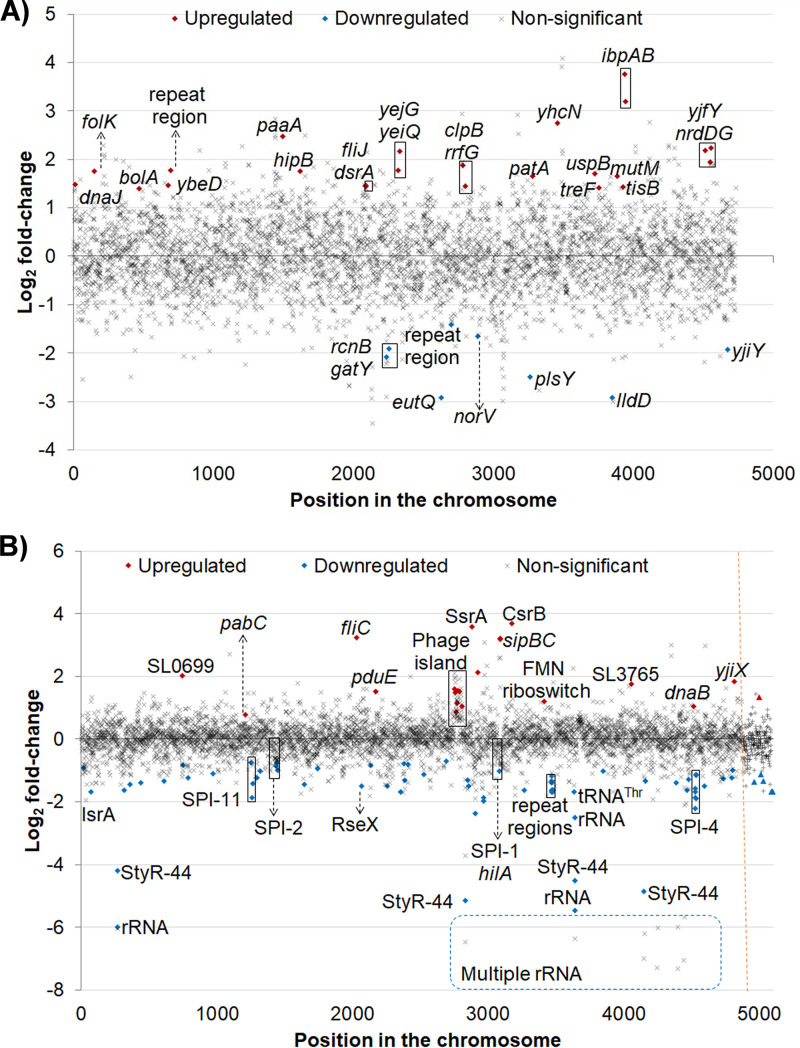
Transcriptional landscape of the E. coli and *S*. Typhimurium AcrB D408A mutants in LB broth in stationary phase. Differentially transcribed genes in the AcrB D408A mutants of E. coli (A) and *S. Typhimurium* (B) are shown. Significantly up- and downregulated genes are shown in red and blue, respectively. Genes with nonsignificant changes in transcription are shown in gray. An orange dotted line denotes the end of chromosomal genes in *S. Typhimurium* SL1344 and the start of plasmid genes.

Compared to those in wild-type E. coli MG1655, a total of 32 genetic elements were differentially transcribed in the E. coli AcrB D408A mutant. Twenty-six of these elements encoded proteins and were mapped to the respective COG class. The remainder were small RNAs (sRNAs), toxin-antitoxin modules, and repeat regions with regulatory function (Fig. 2A).

In *S.* Typhimurium, 109 genetic elements were differentially transcribed in the AcrB D408A mutant. These comprised 41 genes that encoded proteins present in the COG database. The remaining 68 genetic elements mapped to noncoding RNA (ncRNA), toxin-antitoxin modules, repeat regions with regulatory function, and rRNA (Fig. 2B).

**FIG 3:**
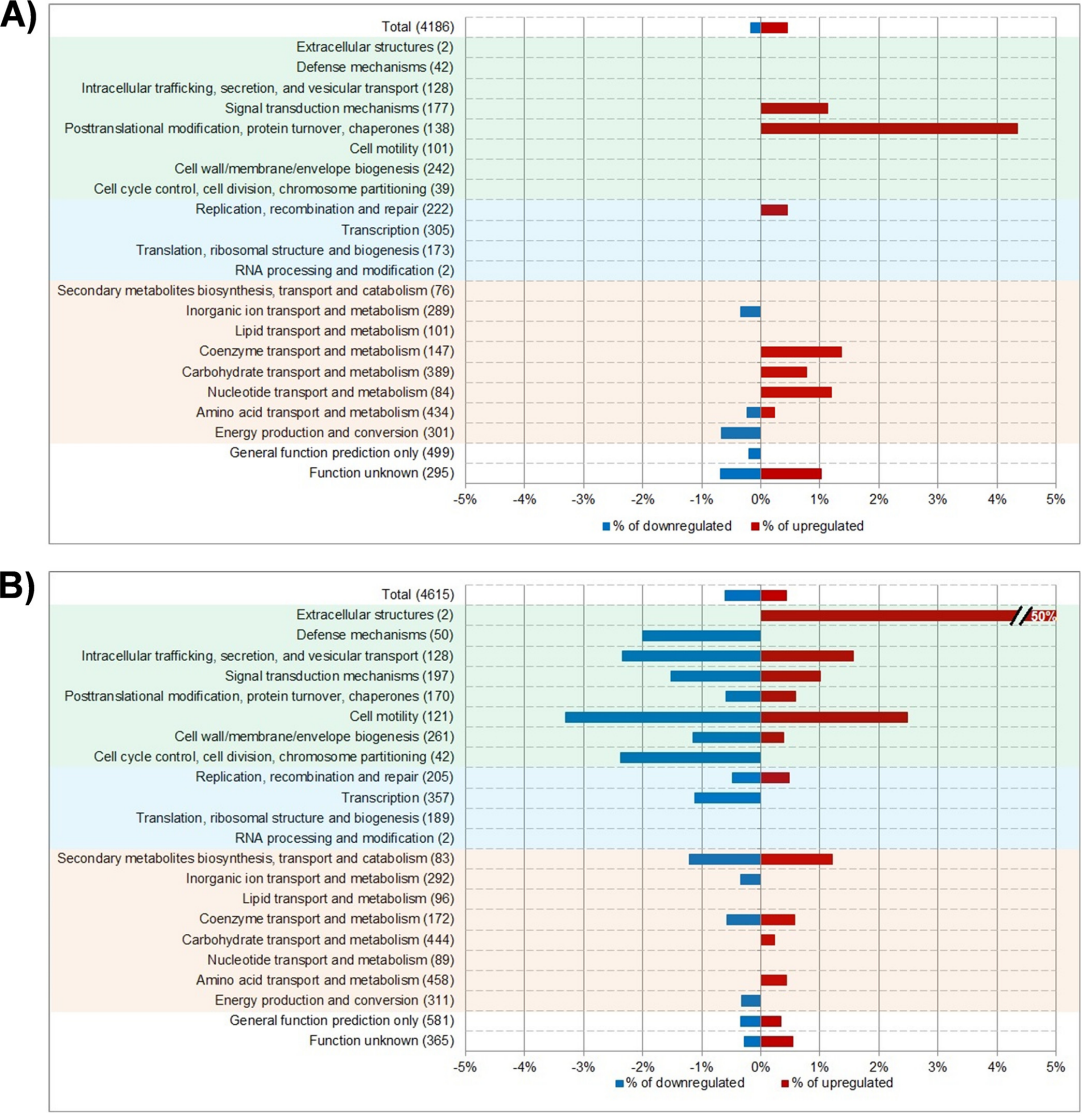
COG classification of differentially transcribed genes in the AcrB D408A mutants of E. coli (A) and *S*. Typhimurium (B) cultured in LB broth. The percentages of downregulated (blue) and upregulated (red) genes in each COG class are shown. The name of each COG class is shown on the left, and the total number of genes in this class are indicated in parentheses. Highlighted in green are the classes involved in “cellular responses and signaling” processes; in blue are those found in the “information storage and processing” process; in orange are classes involved in metabolism.

Comparison of COG analyses revealed that the general pattern of changes in both microorganisms was different, suggesting that the biological impact of the AcrB D408A substitution is different between E. coli and *S.* Typhimurium (Fig. 3) at the genetic and metabolic levels. In general, the loss of AcrB efflux function had a greater effect on the “cellular processes and signaling” COG category in *S.* Typhimurium than in E. coli.

Two classes behaved in a similar manner in both species: (i) the “energy production and conversion” class, which only contained genes that were downregulated, and (ii) the “carbohydrate transport and metabolism” class, which only contained significantly upregulated genes.

## DISCUSSION

The AcrAB-TolC MDR efflux pump is well known for its role in conferring multidrug resistance to Gram-negative bacteria. However, the physiological function of the pump and its native physiological substrates are unknown. To gain insight into this, we studied the endometabolome and exometabolome of E. coli MG1655 and *S.* Typhimurium SL1344, two closely related members of the *Enterobacterales* family with distinct physiology and metabolism (16, 17). An untargeted metabolomics approach was used, which provided putative identification of metabolites to allow their allocation into metabolite classes and facilitate the interpretation of results. When cultured in a minimal medium containing no metabolites (except glucose) such as MOPS, significant changes were observed in the endometabolomes of the strains, although no significant metabolic changes were observed in the exometabolome of the wild type compared to that of its respective isogenic AcrB D408A mutant. We hypothesized that this is a result of growth in minimal medium, where metabolite synthesis by the cell is required, and so efflux of these essential metabolites is not favorable (20). In support of this hypothesis, a recent review suggested that in a time course experiment, the lack of significant changes in the exometabolome could be a consequence of regulation of metabolite secretion mechanisms (12). Based on this proposal, we further hypothesized that, AcrAB-TolC would only export metabolites in a metabolite-rich medium, such as in LB broth. In accordance with this, when E. coli and *S.* Typhimurium were cultured in LB broth, significant changes in metabolite relative concentrations in the exometabolomes of the mutants were observed, suggesting that a rich medium may promote efflux activity. It is also possible that active export of metabolites in minimal medium occurs at an earlier growth phase and that exported metabolites are internalized during stationary phase, where metabolic turnover and overflow metabolism will be reduced.

Despite the lack of differences between the wild-type and mutant exometabolomes in MOPS, loss of AcrB-mediated efflux resulted in changes in the intracellular metabolome (endometabolome) of the bacteria when cultured in MOPS. Two metabolic classes were of particular interest: sphingolipid metabolism and oxidized fatty acid metabolites. Sphingolipids are not known to be synthesized by E. coli or *Salmonella* spp. However, sphingolipid biosynthesis in bacteria is not well characterized, as bacterial enzymes required for sphingolipid biosynthesis cannot be identified by sequence homology to eukaryotic enzymes (21). As proof of principle, it was recently shown that Caulobacter crescentus synthesizes glycosphingolipids under phosphate starvation conditions and not in rich media (22). The cellular origin of the lipids in this study cannot be elucidated, but it is hypothesized that they could derive from the cell membrane. RND pumps have been shown to export precursors of cell wall components in Mycobacterium tuberculosis, Mycobacterium smegmatis, and Corynebacterium glutamicum (23, 24). Additionally, a recent lipidomics study in Acinetobacter baumannii showed that changes in the intracellular concentration of glycerophospholipids, specifically, phosphatidylcholine, were associated with activity of AdeABC and AdeIJK (homologues of AcrAB-TolC in A. baumannii) (25). Glycerophospholipids are found primarily in the membrane (26), further suggesting that RND efflux pumps may be involved in export or regulation of the biosynthesis of membrane components under specific growth conditions.

The analysis of the endometabolome in MOPS minimal medium revealed that there is no overlap between the metabolic profiles of the E. coli and the *S.* Typhimurium AcrB D408A mutants. This was surprising, as we had originally hypothesized that there would be some overlap between the two mutants given the high similarity of AcrB between the two species and the potential of the pump to export the same xenobiotics. However, the absence of overlap between the two *acrB* mutants suggests that the underlying genotypes and metabolism of E. coli and *S.* Typhimurium greatly influences the natural substrate profile of AcrB.

In the exometabolome study in LB broth (a metabolite-rich medium), a greater number of differences between the *acrB* mutant and its respective wild type were observed for *S.* Typhimurium than for E. coli (174 and 11 metabolites, respectively). We hypothesize that this is due to E. coli MG1655 having more metabolite transporters than *S.* Typhimurium SL1344 (27), which can lessen the metabolic burden of losing AcrB efflux activity. Based on the classes observed, E. coli MG1655 harbors 245 membrane transporters from these families, whereas *S.* Typhimurium SL1344 harbors 237 of these transporters (27). The 3% difference in number of transporters may seem small, but it is well documented that the overexpression or deletion of a single efflux pump leads to significant changes in drug resistance phenotypes (28). Therefore, the difference in number of transporters, however small, may have a significant impact in metabolite transport in bacteria. Since 1% of the core E. coli genome is composed of genes coding for efflux pumps (29), we hypothesized that metabolites effluxed through AcrB in E. coli could be exported via other transporters when AcrB is inactive, decreasing the impact of the AcrB D408A substitution on the exometabolome of E. coli. However, the differential gene expression data did not show significant changes in transcription of the other pump genes. This may be because most RND efflux pump and many other transporter genes are transcribed during the exponential growth phase, and RNA sequencing (RNA-seq) was carried out from stationary-phase cultures to match the metabolomics analysis conditions. Although, proteins such as AcrB are stable and have a long half-life well into stationary phase.

An unexpected side observation of the present study was that, unlike in *S.* Typhimurium (18), inactivation of *acrB*/AcrB in E. coli did not affect the efflux of ethidium bromide, yet the strain was hypersusceptible to the dye. It was initially hypothesized that MdtF (27), an RND efflux pump present in E. coli MG1655 and not in *S.* Typhimurium SL1344, was accountable for this phenotype, as overexpression of the pump increases the MIC of ethidium bromide in E. coli (30) and confers innate tolerance to the dye during stationary-phase growth (31). A previous study reported that the maximum induction of *mdtEF* occurs during stationary phase (31). Therefore, we looked for differential expression of *mdtEF* in our RNA-seq data. However, this hypothesis was not supported, as *mdtF* was not differentially transcribed in the E. coli AcrB D408A mutant. Nonetheless, it cannot be ruled out that other efflux pumps and/or porins are responsible for the absence of a significant effect in ethidium bromide efflux in E. coli, and this requires further investigation.

Interestingly, an increase in transcription of multiple regulatory RNAs and noncoding regions was observed in both *acrB* mutants, irrespective of the background. This suggests changes in gene expression networks that could directly or indirectly impact metabolism of the microorganisms in LB broth; for example, lack of AcrB function could cause the accumulation of a metabolite or its biosynthetic intermediates that could potentially inhibit biosynthesis of said metabolite through a negative feedback loop. COG comparison results suggested that the most affected metabolic pathways are associated with carbohydrate usage. A role for AcrB in carbohydrate utilization was previously suggested in a microarray study on an E. coli BW25113 Δ*acrB* mutant after growth in LB broth to exponential growth phase (32). Ruiz and Levy (32) suggested that AcrB was involved in the export of metabolic intermediates from gluconeogenesis as well as cysteine, purine, and enterobactin biosynthesis. However, our exometabolome analysis of LB broth exometabolome analysis did not show significant changes in carbohydrate utilization pathways in E. coli or *S.* Typhimurium. This contrasts a recent study, where untargeted metabolomics was used to demonstrate changes in central carbon metabolism when *acrB* was deleted or overexpressed in E. coli BW25113 grown in the metabolite-rich EZ rich defined medium to exponential growth phase (33). It is possible that changes in carbohydrate utilization were not detected in the current study due to the AcrB D408 mutant producing wild-type levels of protein, and the effects were due to lack of or overexpression of AcrB rather than efflux function, medium composition, and/or growth phase at which the cells were harvested.

The exometabolome analysis of strains grown in LB broth suggested that the biological impact of loss of AcrB-mediated efflux would be less in E. coli than in *S.* Typhimurium. This hypothesis was explored by comparing differential gene transcription profiles of the E. coli and *S.* Typhimurium AcrB D408A mutants. In general, more COG classes were differentially transcribed in *S.* Typhimurium than in E. coli, which supports the hypothesis that AcrB efflux function has a greater impact in *S.* Typhimurium than E. coli when grown in LB broth and during stationary growth phase. An additional hypothesis is that the difference in LB exometabolomes between E. coli and *S.* Typhimurium is associated with the virulence traits of *S.* Typhimurium SL1344, which is virulent in mice and calves (34). In *S.* Typhimurium, loss of the AcrB protein or AcrB-mediated efflux caused attenuation of virulence of the mutants in murine infection models and gentamicin protection assays (18, 35–37). Therefore, it has been proposed that AcrB in *S.* Typhimurium exports virulence-associated molecules. We propose that some of the metabolites found in abundance in the wild-type exometabolome of *S.* Typhimurium are involved in virulence. In LB broth, the metabolite classes which demonstrated higher metabolite relative concentrations in the *S.* Typhimurium wild-type exometabolome (compared to that in mutant exometabolome) were aromatic amino acids and associated metabolites (e.g., tryptophan metabolism), acyl carnitines, and polyamines. Metabolites from these classes have been shown to modulate virulence of S. enterica and other bacteria. Examples of these include indole, a tryptophan-associated metabolite, which attenuates virulence of *S*. Typhimurium, although S. enterica cannot synthesize it (38, 39). Another group of tryptophan metabolism products are quinolones, such as the *Pseudomonas* quinolone signal (PQS), which regulates virulence in P. aeruginosa and precursors of which are exported by RND efflux pumps (40, 41). The acyl carnitine, palmitoylcarnitine, modulates the host immune response during infection with *S.* Typhimurium, by disrupting the structure of mesenteric lymph nodes in a murine infection model (42). Polyamines such as putrescine and spermidine are required for the expression of *S.* Typhimurium virulence factors encoded in *Salmonella* pathogenicity islands 1 and 2 (SPI-1 and SPI-2, respectively) (43). These metabolite classes contain potential candidates to test for virulence-modulating metabolites. Of most note, the involvement of AcrB in export of any of these metabolite classes has not been characterized to date.

The concentration of metabolites detected in the exometabolome relies on the metabolite concentration in the medium at the start of the culture (assuming the metabolite is present in the medium), the rate of influx into the cell defined as the intracellular availability of the metabolite, and the rate of efflux out of the cell into the exometabolome; for example, increased biosynthesis of a compound may result in increased efflux out of the cell. It is difficult in nontargeted metabolomics experiments to differentiate between changes in nutrient influx into the cell from the medium, efflux from the cell into the medium, and changes in intracellular biosynthesis. A time course experiment or a more targeted isotope tracer experiment should help to unravel these complexities. Nonetheless, it was striking that oxidized fatty acids were detected in abundance in the endometabolomes of the E. coli and *S.* Typhimurium AcrB D408A mutants grown in MOPS. This suggests that these are accumulated intracellulary in the absence of AcrB efflux activity. In the exometabolome of the *acrB* mutants grown in LB broth, the relative concentration of oxidized fatty acids was decreased. These observations suggest that oxidized fatty acids are potential native substrates of AcrB in both E. coli and *S.* Typhimurium. This hypothesis is supported by (i) previous reports in which an E. coli MG1655 mutant engineered to produce free fatty acids, TY05, exported the fatty acids via AcrB (10) and that the RND AdeIJK efflux system in Acinetobacter baumannii is implicated in export of host fatty acids (44), and (ii) none of the previous studies with E. coli Δ*acrB* (32, 33) and *S.* Typhimurium AcrB D408A (18) mutants have shown significant changes in fatty acid biosynthetic pathways.

In summary, our study is the first to suggest that oxidized fatty acids are potential native substrates of AcrB in two members of the *Enterobacterales* family and to provide insight into potential virulence-modulating metabolites exported by AcrB in *S.* Typhimurium.

## MATERIALS AND METHODS

### Bacterial strains and growth conditions.

*S.* Typhimurium SL1344 and E. coli K-12 MG1655 were used as genetic backgrounds for the construction of the *acrB* mutants used in this study. The construction of the chromosomal *S.* Typhimurium AcrB D408A mutant was described previously (18, 45). The same method was used for the construction of the chromosomal E. coli AcrB D408A mutant.

All strains were incubated at 37°C in LB broth (Sigma, UK) or MOPS minimal medium with glucose as carbon source (Teknova, USA). When MOPS minimal medium was used, thiamine (Sigma, USA) at a final concentration of 1 μg/ml was added to the medium to facilitate growth of E. coli MG1655. To facilitate growth of *S.* Typhimurium SL1344 in MOPS minimal medium, a final concentration of 25 μM histidine (Sigma, USA) was added. No supplements were added to LB broth.

### Western blot and phenotypic characterization of the E. coli AcrB D408A mutant.

Western blot, determination of MICs of antibiotics and dyes by agar dilution, growth kinetics, ethidium bromide efflux, and Hoechst accumulation assays were carried out as described in by Wang-Kan et al. (18).

### Preparation and analysis of endo- and exometabolomes.

Endo- and exometabolomes were prepared as described previously (46). Samples were analyzed by ultrahigh-performance liquid chromatography-mass spectrometry (UHPLC-MS) at the Phenome Centre Birmingham using a Dionex UltiMate 3000 rapid separation LC system (Thermo Fisher Scientific, MA, USA) coupled with and an electrospray Q Exactive Focus mass spectrometer (Thermo Fisher Scientific, MA, USA). Detailed information on preparation of samples and analysis can be found in Text S1 in the supplemental material.

10.1128/mBio.00109-21.9 TEXT S1 Supplementary materials and methods. Download Text S1, DOCX file, 0.03 MB. Copyright © 2021 Wang-Kan et al. 2021 Wang-Kan et al. https://creativecommons.org/licenses/by/4.0/ This content is distributed under the terms of the Creative Commons Attribution 4.0 International license.

### RNA sequencing and data analysis.

RNA sequencing was carried out as previously described (18, 47). In brief, an overnight culture of each strain was diluted 1:100 in LB broth. Cultures were incubated at 37°C, shaking at 200 rpm, for 10 h to allow bacteria to reach stationary phase. An aliquot of 2 ml of each culture was quenched in 2/5 of ice-cold phenol-ethanol as described by Kroger et al. (48). Pellets were collected by centrifugation, and RNA was extracted using the RNeasy minikit (Qiagen, USA) as instructed by the manufacturer. Library construction and sequencing was provided by the Beijing Genomics Institute, and data were analyzed as described previously (18, 47). COG databases were obtained from MicroScope (49).

### Data availability.

Spectral files are available on Metabolights, under accession number MTBLS2263. RNA sequencing data are available on ArrayExpress, under accession number E-MTAB-8352.
