# Supplementary figures and images for: Metabolomics Reveal Potential Natural Substrates of AcrB in Escherichia coli and Salmonella enterica Serovar Typhimurium
Source: mBio. 2021 Mar 30;12(2):e00109-21. doi: 10.1128/mBio.00109-21 (PMC8092203; doi:10.1128/mBio.00109-21)

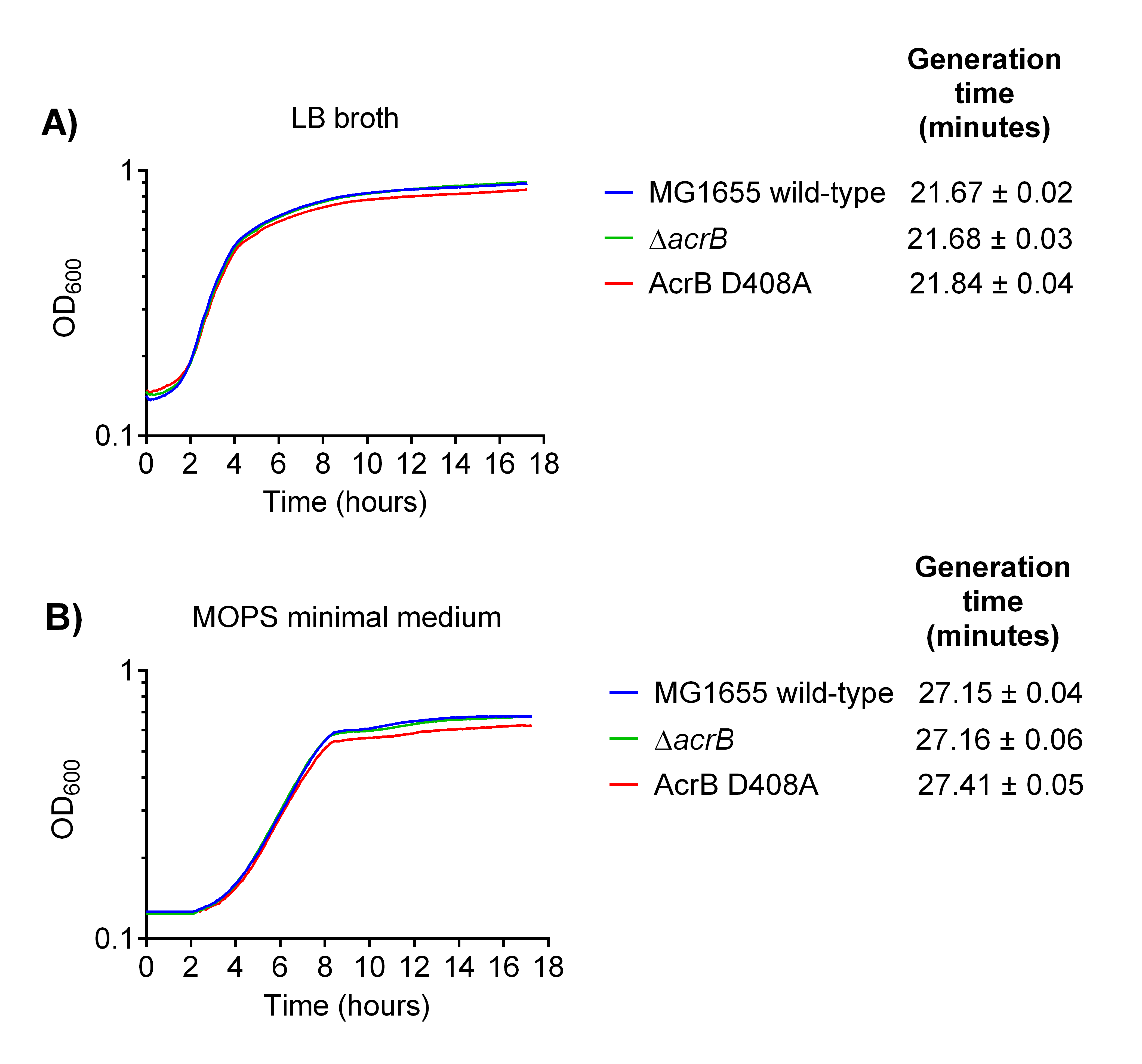

Supplement: FIG S2 [file mBio.00109-21-sf002.tif]

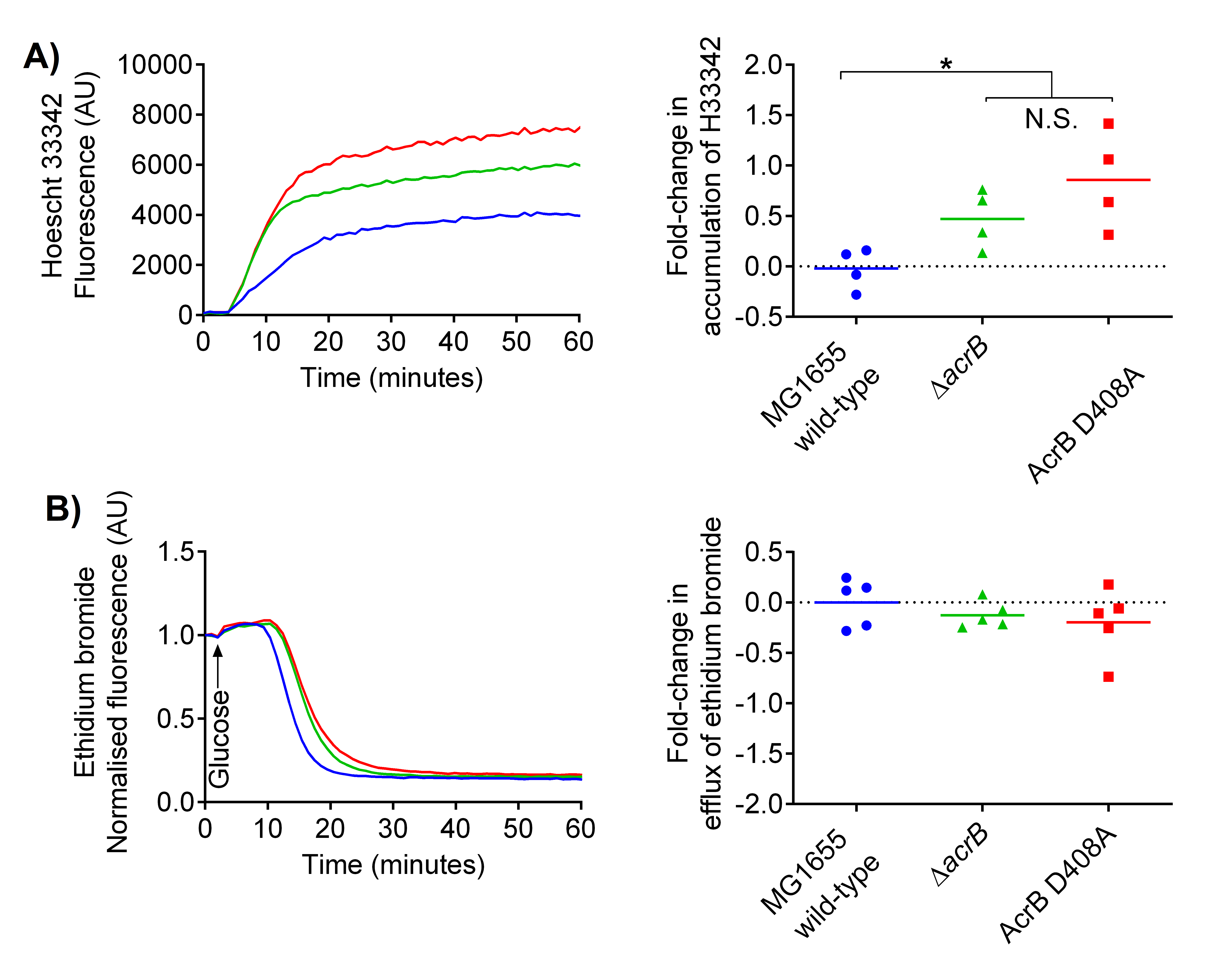

Supplement: FIG S3 [file mBio.00109-21-sf003.tif]
