# Supplementary material for: Metabolomics Reveal Potential Natural Substrates of AcrB in Escherichia coli and Salmonella enterica Serovar Typhimurium
Source: mBio. 2021 Mar 30;12(2):e00109-21. doi: 10.1128/mBio.00109-21 (PMC8092203; doi:10.1128/mBio.00109-21)
Supplement: TABLE S1 [file mBio.00109-21-st001.docx]

**Table S1.** **Minimum inhibitory concentrations of AcrB substrates for *E. coli* MG1655 and its respective *acrB* mutants.**

| Strain | MIC (µg/mL) | | | | | | | | | | | |
| --- | --- | --- | --- | --- | --- | --- | --- | --- | --- | --- | --- | --- |
|  | CIP | NAL | NOV | CHL | TET | MIN | FUS | OX | ERY | ACR | ETBR | RHO |
| MG1655 | 0.01 | 8 | 1024 | 4 | 4 | 4 | >1024 | 512 | 256 | 128 | 512 | 512 |
| ∆*acrB* | <0.008 | 2 | 16 | 1 | 1 | 0.5 | 1024 | 4 | 64 | 8 | 32 | 8 |
| AcrB D408A | <0.008 | 2 | 16 | 1 | 1 | 0.5 | 1024 | 4 | 64 | 8 | 32 | 8 |

CIP, ciprofloxacin; NAL, nalidixic acid; NOV, novobiocin; CHL, chloramphenicol; TET, tetracycline; MIN, minocycline; FUS, fusidic acid; OX, oxacillin; ERY, erythromycin; ACR, acriflavine; ETBR, ethidium bromide; RHO, rhodamine 6G. Agar dilution MICs were determined four times. Mode values are shown.
