# Supplementary material for: Metabolomics Reveal Potential Natural Substrates of AcrB in Escherichia coli and Salmonella enterica Serovar Typhimurium
Source: mBio. 2021 Mar 30;12(2):e00109-21. doi: 10.1128/mBio.00109-21 (PMC8092203; doi:10.1128/mBio.00109-21)
Supplement: TEXT S1 [file mBio.00109-21-s0001.docx]

**Text S1. Supplementary materials and methods.**

## Preparation and analysis of endo- and exometabolome

Endo- and exometabolomes were prepared as described previously (1). In brief, to obtain sufficient cell density to carry out metabolomics experiments, ten overnight cultures per strain were prepared in LB broth or MOPS minimal medium. Viable counts were carried out to ensure that the cultures contained the same amount of bacteria so that comparisons could be made. Cell-free culture supernatants were obtained from the overnight cultures. A 0.22 μm pore, PVDF membrane syringe filter was used for filter-sterilisation (Starlabs, UK). Aliquots of 1 mL were stored at -80°C, in 1.5 mL microcentrifuge tubes (Eppendorf, Germany). On the day of analysis, exometabolome samples were thawed on wet ice and vortexed briefly. To create the pooled QC sample, 50 μL from each sample was pooled and vortexed, and then split into multiple 50 μL aliquots and kept on wet ice. To prepare samples for UHPLC-MS analysis, 50 μL exometabolome sample or QC was mixed with 150 μL of ice-cold acetonitrile (LC-MS grade, VWR), vortexed (30 s), centrifuged (21,000-g, 20 min, 4°C). 100 μL of the supernatant was loaded into a low recovery volume LC autosampler vial with glass insert (VI-04-12-02RVG, Chromatography Direct, UK). Extraction blank samples were prepared as above but substituting the 50 μL exometabolome aliquot with 50 μL water (LC-MS grade, VWR).

## Preparation of endometabolome

The procedure described by C. L. Winder et al. (1) with some modifications as described was applied. In brief, an aliquot of 1 mL of each one of ten overnight cultures grown in MOPS minimal medium were added to an equal volume of 60% aqueous methanol at -40°C. Bacterial pellets from quenched cultures were harvested by centrifugation at 3,500X*g* for 20 minutes, at 4°C. To extract metabolites, ice cold 1.5 mL acetonitrile/water (75/25, v/v; LC-MS grade, VWR) was added to the frozen bacterial pellets and mixed with a pipette to resuspend bacteria. The sample was frozen on dry ice, then allowed to thaw on wet ice. The sample was then homogenised using a bead-based homogeniser (Precellys24 and VK05 tubes containing glass beads for micro-organism lysis, Stretton Scientific, UK) for 2 x 10 s bursts of 6400 rpm. Samples were centrifuged (21,000-g, 20 min, 4°C) and 900 μL of the supernatant was removed and dried in a SpeedVac sample concentrator (Savant SPD111V230, Thermo Fisher Scientific). A further 400 μL of the supernatant was removed from each sample and pooled (vortex 30 s) to create the pooled QC. The pooled QC was split into 900 μL aliquots and dried in a SpeedVac. Extraction blank samples were prepared as above but without the bacterial sample. All dried extracts were stored at -80⁰C until analysis. On the day of analysis, samples were resuspended in 150 μL ice cold acetonitrile/water (75/25, v/v), vortexed (30 s) and centrifuged (21,000-g, 20 min, 4°C). 100 μL of the supernatant was loaded into a low recovery volume LC autosampler vial with glass insert (VI-04-12-02RVG, Chromatography Direct, UK).

## Analysis of the composition of the endo- and exometabolome

Samples were analysed by UHPLC-MS (ultra-high performance liquid chromatography-mass spectrometry) at the Phenome Centre Birmingham using a Dionex UltiMate 3000 Rapid Separation LC system (Thermo Fisher Scientific, MA, USA) coupled with and electrospray Q Exactive Focus mass spectrometer (Thermo Fisher Scientific, MA, USA) by two separate assays, Assay 1 and Assay 2. Metabolite concentrations in the nanomolar to millimolar concentration range are typically detected applying this untargeted metabolomics approach. Assay 1 was a UHPLC-MS HILIC assay, that used an Accucore-150-Amide-HILIC column (100 x 2.1 mm, 2.6 μm, Thermo Fisher Scientific, MA, USA). Data collection was carried out in positive and negative ionisation modes separately to maximise metabolite detection. Mobile phase A was 10 mM ammonium formate and 0.1% formic acid in 95% acetonitrile/water, and mobile phase B was 10 mM ammonium formate and 0.1% formic acid in 50% acetonitrile/water. The gradient was as follows: t=0.0, 1% B; t=1.0, 1% B; t=3.0, 15% B; t=6.0, 50% B; t=9.0, 95% B; t=10.0, 95% B; t=10.5, 1% B; t=14.0, 1% B. All changes were linear (curve = 5) and the flow rate was 0.50 mL/min. Column temperature was 35 °C and injection volume was 2 μL. Data were acquired in positive and negative ionisation modes separately (70 – 1050 m/z) with a resolution of 70,000 (FWHM at m/z 200). Ion source parameters: Sheath gas = 53 arbitrary units, Aux gas = 14 arbitrary units, Sweep gas = 3 arbitrary units, Spray Voltage = 3.5kV (positive ion) / 2.7kV (negative ion), capillary temp. = 269 °C (positive ion)/320 °C (negative ion), aux. gas heater temp. = 438 °C (positive ion)/320 °C (negative ion). Data dependent MS2 in ‘Discovery mode’ was applied to three QC samples over three mass ranges (70 – 200 m/z; 200 – 400 m/z; 400 – 1000 m/z) using following settings: resolution = 17,500; Isolation width = 3.0 m/z; stepped normalised collision energies = 25, 60, 100%. Assay 2 was a UHPLC-MS C_18_ aqueous reversed phase assay, that used either a Waters HSS T3 column (*S.* Typhimurium cultured in LB medium; 100 x 2.1 mm, 1.8 μm, Waters Ltd, UK) or a Hypersil Gold aQ column (*S.* Typhimurium cultured in MOPS medium and *E. coli* cultured in MOPS and LB medium; 100 x 2.1 mm, 1.9 μm, Thermo Fisher Scientific, USA). Data collection was carried out in positive and negative ionisation modes separately to maximise metabolite detection. Mobile phase A was water with 0.1% formic acid and mobile phase B was acetonitrile with 0.1% formic acid. The gradient was as follows: t=0.0, 1% B; t=0.5, 1% B; t=2.0, 50% B; t=9.0, 99% B; t=10.0, 99% B; t=10.5, 1% B; t=15.0, 1% B. All changes were linear (curve = 5) and the flow rate was 0.30 mL/min. Column temperature was 45 °C and injection volume was 2 μL. Data were acquired in positive and negative ion-isation modes separately (100 – 1500 m/z) with a resolution of 70,000 (FWHM at m/z 200). Ion source parameters: Sheath gas = 48 arbitrary units, Aux gas = 11 arbitrary units, Sweep gas = 2 arbitrary units, Spray Voltage = 3.5kV (positive ion) / 2.5kV (negative ion), Capillary temp. = 256 °C, Aux gas heater temp. = 413°C. Data dependent MS2 in ‘Discovery mode’ was applied to three QC samples over three mass ranges (100 – 300 m/z; 300 – 600 m/z; 600 – 1500 m/z) using following settings: resolution = 17,500; Isolation width = 3.0 m/z; stepped normalised collision energies = 20, 50, 80%.

ProteoWizard software (2) was used to convert the acquired raw data to the mzML file format. Deconvolution was performed with XCMS software according to the parameters established by W. B. Dunn *et al*. (3). PUTMEDID-LCMS workflows operating in the Taverna workflow environment (4) was used to provide putative annotation of metabolites. A 5 ppm mass error and a retention time range of 2 s in feature grouping and molecular formula and metabolite matching was applied. All molecules were annotated according to guidelines for reporting of chemical analysis results as proposed in Metabolomics Standards Initiative level 3 (5). Statistical analysis was performed applying the web-based platform MetaboAnalyst (6) applying no missing value imputation, normalisation to total peak area per sample and no scaling or transformation. Non-parametric Mann-Whitney U test and Kruskal-Wallis one-way ANOVA tests were applied. Fold changes were calculated using the mean response for each biological class.

**References**

1. Winder CL, Dunn WB, Schuler S, Broadhurst D, Jarvis R, Stephens GM, Goodacre R. 2008. Global metabolic profiling of *Escherichia coli* cultures: an evaluation of methods for quenching and extraction of intracellular metabolites. Anal Chem 80:2939-48.

2. Chambers MC, Maclean B, Burke R, Amodei D, Ruderman DL, Neumann S, Gatto L, Fischer B, Pratt B, Egertson J, Hoff K, Kessner D, Tasman N, Shulman N, Frewen B, Baker TA, Brusniak M-Y, Paulse C, Creasy D, Flashner L, Kani K, Moulding C, Seymour SL, Nuwaysir LM, Lefebvre B, Kuhlmann F, Roark J, Rainer P, Detlev S, Hemenway T, Huhmer A, Langridge J, Connolly B, Chadick T, Holly K, Eckels J, Deutsch EW, Moritz RL, Katz JE, Agus DB, MacCoss M, Tabb DL, Mallick P. 2012. A cross-platform toolkit for mass spectrometry and proteomics. Nat Biotechnol 30:918-920.

3. Dunn WB, Broadhurst D, Brown M, Baker PN, Redman CW, Kenny LC, Kell DB. 2008. Metabolic profiling of serum using Ultra Performance Liquid Chromatography and the LTQ-Orbitrap mass spectrometry system. J Chromatogr B Analyt Technol Biomed Life Sci 871:288-98.

4. Brown M, Wedge DC, Goodacre R, Kell DB, Baker PN, Kenny LC, Mamas MA, Neyses L, Dunn WB. 2011. Automated workflows for accurate mass-based putative metabolite identification in LC/MS-derived metabolomic datasets. Bioinformatics 27:1108-12.

5. Sumner LW, Amberg A, Barrett D, Beale MH, Beger R, Daykin CA, Fan TWM, Fiehn O, Goodacre R, Griffin JL, Hankemeier T, Hardy N, Harnly J, Higashi R, Kopka J, Lane AN, Lindon JC, Marriott P, Nicholls AW, Reily MD, Thaden JJ, Viant MR. 2007. Proposed minimum reporting standards for chemical analysis Chemical Analysis Working Group (CAWG) Metabolomics Standards Initiative (MSI). Metabolomics 3:211-221.

6. Pang Z, Chong J, Li S, Xia J. 2020. MetaboAnalystR 3.0: Toward an Optimized Workflow for Global Metabolomics. Metabolites 10.
